# *Anemopsis californica* Attenuates Photoaging by Regulating MAPK, NRF2, and NFATc1 Signaling Pathways

**DOI:** 10.3390/antiox10121882

**Published:** 2021-11-25

**Authors:** Quynh T. N. Nguyen, Minzhe Fang, Nhung Quynh Do, Jeehaeng Jeong, Sarang Oh, Shengdao Zheng, Minseon Kim, Junhui Choi, Seojun Lim, Tae Hoo Yi

**Affiliations:** 1 Graduate School of Biotechnology, Kyung Hee University, 1732 Deogyeong-daero, Giheung-gu, Yongin-si 17104, Korea; quynhnguyen@khu.ac.kr (Q.T.N.N.); mincheol1030@khu.ac.kr (M.F.); quynhnhung96@khu.ac.kr (N.Q.D.); sdjeong0719@khu.ac.kr (S.Z.); dbs03067@khu.ac.kr (M.K.); junhi4703@khu.ac.kr (J.C.); ongane1@khu.ac.kr (S.L.); 2 Snow White Factory, 807 Nonhyeon-ro, Gangnam-gu, Seoul 06032, Korea; dbensk0205@khu.ac.kr (J.J.); espritmj@khu.ac.kr (S.O.)

**Keywords:** *Anemopsis californica*, oxidative stress, photoaging, nuclear factor erythroid 2–related factor 2 (NRF2), nuclear factor of activated T-cells (NFAT)

## Abstract

Long-term exposure of the skin to solar radiation causes chronic inflammation and oxidative stress, which accelerates collagen degradation. This contributes to the formation of wrinkles and dark spots, skin fragility, and even skin cancer. In this study, *Anemopsis californica* (AC), a herb from North America that is well known for treating microorganism infection and promoting wound healing, was investigated for its photoprotective effects. The biological effects of AC were studied on two in vitro models, namely, lipopolysaccharide (LPS)-induced macrophages and ultraviolet B (UVB)-irradiated dermal fibroblasts, to characterize its underlying molecular mechanisms. The results showed that AC decreased the mRNA levels of inflammatory mediators in sensitized macrophages, including cytokines, inducible nitric oxide synthase (iNOS), and cyclooxygenase (COX-2). Moreover, AC alleviated UVB-induced photoaging in dermal fibroblasts by restoring procollagen synthesis. This resulted from the regulation of excessive reactive oxygen species (ROS) by AC, which was mediated by the activation of the antioxidative system nuclear factor erythroid 2-related factor 2 (NRF2). AC also alleviated oxidative stress and inflammatory responses by inhibiting the phosphorylation of mitogen-activated protein kinase (MAPK) and interfering with the nuclear translocation of the immune regulator nuclear factor of activated T-cells 1 (NFATc1). In conclusion, the protective effects of AC on skin cellular components suggested that it has the potential for use in the development of drugs and cosmetics that protect the skin from UVB-induced chronic inflammation and aging.

## 1. Introduction

As the primary defense system of the body, the skin directly undergoes physiological changes caused by ambient factors such as pollution, bacteria, and chronic light exposure. Solar radiation, which contains >10% ultraviolet (UV) light, is considered to be a major cause of skin photoaging, which is characterized by rough wrinkles, dark spots, and dryness [1]. Although atmospheric ozone absorbs UVC radiation (100–280 nm), UVA (315–400 nm) and UVB (280–315 nm) radiation can reach the earth’s surface and directly penetrate layers of the skin [2]. The more energetic radiation, UVB, also called “burn rays,” reaches the epidermis unimpeded and partly reaches the upper dermis [2]. Histological studies of the skin have reported an increase in mononuclear cell infiltration and degraded elastic fibers under conditions of repeated exposure to sunlight, when compared with shielded skin [3]. Exposure to UVB leads to the recruitment of macrophages to remove the damaged skin cells with oxidized surface lipids [4]. Activated macrophages release cytokines such as interleukins (ILs) and tumor necrosis factor alpha (TNF-α), as well as inflammatory molecules, including nitric oxide (NO) and prostaglandin E_2_ (PGE_2_), leading to chronic inflammation and thus triggering inflamm-aging [5]. These molecules stimulate the expression of matrix metalloproteinases (MMPs) to disrupt the collagen fibers that are present in the extracellular matrix (ECM) of the dermal skin layer, supporting the migration of macrophages through the dermis to injured sites [3]. Therefore, it is important to manage the release of inflammatory mediators to alleviate immunological responses in photoaged skin.

UVB irradiation also induces oxidative stress in dermal fibroblasts, which is characterized by the overproduction of reactive oxygen species (ROS) [6]. ROS accelerate the phosphorylation of mitogen-activated protein kinase (MAPK) subunits, thus causing the activation of transcription factor activating protein-1 (AP-1) to initiate the transcription of collagenase MMPs [6]. It was also reported that UV radiation downregulated the expression of the transforming growth factor beta (TGF-β) receptor, which is involved in the synthesis of procollagen type I, by the production of ROS [7]. In addition, expression of the *TGF-β1* gene has been inhibited by a blockade of the Smad signaling pathway by AP-1 [8]. Thus, the ROS level needs to be decreased in order to sustain collagen synthesis, which further maintains the integrity and elasticity of the skin.

To defend against the excessive production of ROS, cells can activate the antioxidative system called nuclear erythroid 2-related factor (NRF2) [9]. A high level of intracellular ROS frees cytosolic NRF2 from its inhibitor, Kelch like-ECH-associated protein 1 (KEAP1), allowing it to translocate to the nucleus [9]. NRF2 initiates the transcription of cytoprotective molecules, such as NAD (P)H quinone oxidoreductase-1 (NQO1) and heme oxygenase-1 (HO-1), which removes quinones from biological systems as a detoxification reaction and cleaves the oxidative radicals of heme groups, respectively [10]. Moreover, it was reported that UVB irradiation inhibited the expression of the dihydrolipoamide dehydrogenase (DLD) protein in the tricarboxylic acid cycle [11]. DLD not only associates with α-keto acid dehydrogenase, a regulator of ROS, but also metabolizes α-lipoic acid, which activates two cytoprotective proteins, NRF2 and HO-1 [12]. In addition, under hypoxic conditions and UVB exposure, hypoxia-inducible factor 1 alpha (HIF1-α) is stabilized by NRF2 activation, binds to hypoxia-responsive elements in the nucleus, and initiates the transcription of target genes such as HO-1 [13]. Therefore, regulation of the NRF2 signaling pathway has the potential to alleviate UVB-induced oxidative stress.

Oxidative stress also undergoes a bidirectional interaction with calcium signaling, in which ROS mediate calcium signaling, while calcium influx accelerates the activity of ROS-generating enzymes and the synthesis of free radicals [14]. Calcineurin/nuclear factor of activated T cells (NFAT) is composed of transcription factors which are regulated by calcium signaling [15]. NFAT was originally indicated to function as a transcription factor that regulated cytokine expression by associating with the IL-2 promoter, following T-cell activation [15]. However, recent research has indicated that NFAT signaling can regulate not only T cells, but also other immune and non-immune cells. NFAT was reported to be an emerging target for controlling photodamage by UV irradiation. A study by Masaki indicated that the increase of ROS formation in UVB-exposed keratinocytes was accompanied by an immediate elevation of the intracellular calcium level [16]. In addition, Mazière et al. reported that the exposure of fibroblasts to UVA induced the dephosphorylation of nuclear NFATc1, allowing its translocation to the nucleus for transcription initiation [17]. Moreover, Flockhart et al. showed increases in NFAT transcriptional activity and the nuclear localization of NFATc1 in UV-irradiated keratinocytes [18]. NFAT is involved in the mRNA synthesis of inflammation-related genes, including IL-6, TNF-α, PGE_2_, COX-2, and iNOS [19,20]. Furthermore, NFATs have been reported to be pro-invasion transcription factors that upregulate the production of MMPs during tumor invasion [21].

*Anemopsis californica* (AC), belonging to the Saururaceae family, predominantly inhabits southwestern North America [22]. The root powder of AC can be used as a spice and the seeds can be utilized to make bread and various dishes. AC tea and tincture are also widely known as abundant sources of antioxidants and phytoflavanoids [23]. Preliminary analysis of the phytochemical composition of the ethanolic extracts of AC showed that they included tannins, steroids, coumarins, flavonoids, and phenols [24]. In addition, the isolation of active compounds from AC extracts have resulted in the identification of sesamin and asarinin [22]; meanwhile, the most abundant volatile components of AC essential oil were shown to be methyl eugenol (57%), α-pinene (11.7%), elemicin (13.2%), and piperitone (16.2%) [23], which have been well studied for their antioxidant and anti-aging properties [25,26,27]. Studies also confirmed the antioxidative [28], anti-bacterial [22], and anti-cancer [29] effects of AC essential oil and plant extract. However, there have been limited studies on the application of AC in the treatment of skin diseases.

This study was established to investigate the protective effects of AC against LPS-stimulated macrophages (Raw264.7 cells) and UVB-irradiated dermal fibroblasts (HDF cells). This work demonstrated that AC treatment downregulated the LPS-induced mRNA expression of IL-1β, IL-6, TNF-α, iNOS, and COX-2. In addition, AC exerted UVB-induced ROS overproduction, resulting in the restoration of procollagen production. In particular, AC reduced the ROS-triggered phosphorylation of MAPK subunits, leading to a decrease in MMP production and an increase in TGF-β1 expression. AC also activated the NRF2 signaling pathway for the detoxification of excess ROS formation. Finally, AC was found to prevent the nuclear translocation of NFATc1, suggesting that it might moderate inflammatory responses during radiation exposure. These results highlight the potential of AC for the production of drugs and cosmetics for photoaged skin.

## 2. Materials and Methods

### 2.1. Materials

*Anemopsis californica* (AC) leaf powder was purchased from Ecuadorian Rainforest, LLC (Clifton, NJ, USA). Dulbecco’s modified Eagle’s medium (DMEM), fetal bovine serum (FBS), and penicillin–streptomycin were supplied by Gibco RBL (Grand Island, NY, USA). Standard tannic acid, chlorogenic acid, apigenin, positive control ascorbic acid, dexamethasone, and tacrolimus, as well as 5-diphenyltetrazolium bromide (MTT), were purchased from Sigma-Aldrich (St. Louis, MO, USA). An ELISA kit for procollagen type 1 was purchased from Takara (procollagen type 1 C-Peptide ELISA Kit; Takara Bio, Otsu, Japan). Human total MMP-1 and human total MMP-3 ELISA kits were purchased from R&D Systems (Minneapolis, MN, USA). Organic solvents were purchased from Samchun Chemical (Seoul, Korea) and Daejung Chemical & Metal (Siheung, Korea). Inorganic salts were purchased from Sigma-Aldrich. Silica gel was purchased from Merck (Kenilworth, NJ, USA). The primary and secondary antibodies were obtained from Cell Signaling Technology (Beverly, MA, USA), Santa Cruz Biotechnology (Santa Cruz, CA, USA), and Bio-Rad Laboratories, Inc. (Hercules, CA, USA).

### 2.2. Sample Preparation

A weight of 100 g AC was extracted in 500 mL of 70% ethanol and shaken, using a Twist shaker (Daihan Scientific Co., Ltd., Seoul, Korea) for 24 h at room temperature. The extraction was replicated thrice. The extracts were collected and subsequently filtered using filter paper (Whatman, Maidstone, Knent, UK). The sample was concentrated by rotary vacuum evaporation (EYELA WORLD–Tokyo Rikakikai Co., LTD., Tokyo, Japan) at 40 °C. The extract yield was 15.33 ± 0.38%.

### 2.3. Total Phenolic, Flavonoid, and Tannin Contents

The total phenolic content of the AC extract was examined based on the Folin-Ciocalteu colorimetric method [24]. Briefly, either standard gallic acid (6.25–100 µg/mL) or plant extract, was reacted with 1M Folin-Ciocalteu reagent for 15 min. Then, 0.7 M sodium carbonate in NaOH was added, and the mixture was incubated for 1 h. The absorbance value was measured at a wavelength of 625 nm.

The total flavonoid content of the AC extract was quantified based on the aluminum chloride colorimetric method [25]. 50 mg/mL of sodium nitrate was mixed with either standard quercetin (0.03125–1 mg/mL) or plant extract. After incubation for 5 min, aluminum chloride was reacted with the mixture for an additional 6 min. Finally, 1M sodium hydroxide was added and incubated for 40 min. Optical density was determined at a wavelength of 450 nm.

The total tannin content was evaluated based on a HCl–vanillin assay [26]. Either standard catechin (0–900 µg/mL) or plant extract was reacted with 4% vanillin in methanol, following by an addition of 32% sulfuric acid. After 15 min of incubation, the plate was read at 450 nm.

The measurement was performed by a microplate reader (Molecular Devices FilterMax F5; San Francisco, CA, USA). The total phenols, flavonoids, and tannins were presented as gallic acid, quercetin, and catechin equivalents in mg per gram of plant extract, respectively.

### 2.4. HPLC Analysis

The plant extract was prepared in 50% methanol at a concentration of 2 mg/ml. Serial dilutions (2.5, 25, 125, 250, 500, and 1000 µg/mL) of standard compounds (tannic acid, chlorogenic acid, and apigenin) were prepared in methanol. High-performance liquid chromatography (HPLC) was performed on a Dionex Chromelon TM chromatography data system with P580 and UVD100 detectors (Thermo Fisher Scientific Inc., Waltham, MA, USA). Chromatographic separation was performed on a Discovery C_18_ (250 × 4.6 mm, 5-µm particle size). Column temperature was 25 °C; flow rate was 1.0 mL/min; injected volume was 10 µL. The condition for chromatographic separation was described in Supplementary Materials Table S1. The chemical content was quantified by determining the area of the peak in the HPLC analysis, following the formula below:

$$Content\;\left(g/100\right)=\left(\frac{sample\;area}{standard\;area}\right)\times \left(\frac{sample\;dilution\;volume}{standard\;dilution\;volume}\times dilution\;factor\right)\times \left(\frac{sample\;amount}{standard\;amount}\right)\times 100$$

### 2.5. 2,2-Diphenyl-1-Picrylhdrazyl Radical Scavenging Activity

The antioxidant effects of the AC extract on 2,2-diphenyl-1-picrylhdrazyl (DPPH, PubChem CID: 2375032) was examined. Various concentrations of AC (1–250 µg/mL) were tested. Ascorbic acid was used as the positive control. The 0.2 mM DPPH in 100% methanol solution was prepared. An aliquot of a 40 µL sample was reacted with 160 µL of DPPH solution, followed by dark incubation at 37 °C for 30 min. The optical density was determined at a wavelength of 595 nm. The inhibitory effect of the sample was assessed by using the following formula:

$$\mathrm{DPPH}\;\mathrm{radical}\;\mathrm{inhibition}\;(\%)=\frac{{(OD}_{0}-{OD}_{x})}{{OD}_{0}}\times 100$$

OD_0_: Optical density of negative control
OD_x_: Optical density of the sample

### 2.6. 2,2’-Azino-Bis (3-Ethylbenzothiazoline-6-Sulfonic Acid) (ABTS) Radical Scavenging Activity

The antioxidant effects of the AC extract on ABTS (ABTS, PubChem CID: 5464076) was detected. Various concentrations of AC (1–250 µg/mL) were tested. Ascorbic acid was used as positive control. A solution of ABTS was made from the reaction of a 2.5 mM ABTS solution with 1 mM 2,2’-azobis (2-amidinopropane) dihydrochloride (AAPH) and 150 mM sodium chloride. Then, the solution was incubated at 70 °C for 30 min. In each well of a 96-well plate, an aliquot of a 4 µL sample was reacted with 196 µL of ABTS solution, followed by dark incubation at 37 °C for 10 min. The optical density was determined at a wavelength of 405 nm. The inhibitory effect of the sample was assessed by using the following formula:

$$\mathrm{ABTS}\;\mathrm{radical}\;\mathrm{inhibition}\;(\%)=\frac{{(\mathrm{OD}}_{0}-{\mathrm{OD}}_{x})}{{\mathrm{OD}}_{0}}\times 100$$

OD_0_: Optical density of negative control
OD_x_: Optical density of the sample

### 2.7. Cell Culture and Treatment

Murine macrophage Raw264.7 cells were provided by the Korean Cell Bank (Seoul, Korea). Normal adult human primary dermal fibroblasts (HDF) (ATCC PCS-201-012) were purchased from ATCC (Manassas, VA, USA). The cells were grown in an incubator at 37 °C under a humidified atmosphere containing 5% CO_2_. A DMEM medium, supplemented with 10% heat-inactivated FBS and 1% antibiotics and antimycotic solution, was used for cell culture.

To induce an inflammatory response, Raw264.7 cells were sensitized with 1 µg/mL LPS at a cell confluence of 80% for 24 h. An aliquot of 10 µM dexamethasone (positive control) or AC extract (1–50 µg/mL) was diluted in a serum-free medium and supplemented for the same time as the LPS treatment.

To mimic the photoaging process, after HDF cells had reached 80% cell confluence, cell plates with a closed lid were exposed to UVB (144 mJ/cm^2^) radiation using a UVB irradiation machine (Bio-Link BLX-312; Vilber Lourmat GmbH, France). Irradiance (0.1 mW/cm^2^) was measured using a UVB photometer (IL1700 Re-search Radiometer/Photometer; International Light, Peabody, MA, USA). Then, cells were rinsed thrice with warm 1X PBS to remove apoptotic cells. Subsequently, fresh serum-free media containing 10 µM ascorbic acid (positive control) or three doses of AC (1–50 µg/mL) were added to each plate for incubation.

### 2.8. MTT Assay

After 24 h of treatment with LPS, or 72 h of treatment with UVB, 1 mg/mL MTT was added to the cell culture and then incubated for 3 h. After incubation, the medium was discarded, followed by the addition of DMSO to solubilize formazan. The optical density was recorded at a wavelength of 595 nm.

### 2.9. NO Assay

NO production was measured in LPS-induced Raw264.7 cells. Raw264.7 cells were seeded at a density of 1 × 10^6^ cells/mL in 96-well cell culture plates (SPL Life Sciences Co., Ltd., Gyeonggi, Korea) and were incubated for 24 h. 24 h after LPS sensation, the secretion of NO was quantified in the cell culture supernatant. A volume of 100 µL of cell culture supernatant was reacted with 100 µL of Griess reagent, which is a mixture of 1% sulfanilamide in 5% phosphoric acid and 0.1% naphthylethylenediamine dihydrochloride (1:1 ratio). Then, the plate was incubated for 10 min at 37 °C. The absorbance density was measured at 595 nm.

### 2.10. ROS Assay

Intracellular ROS levels were measured in UVB-exposed HDF cells. After 24 h of the sample treatment and sensitizer exposure, the supernatant was discarded, and the cells were incubated with 30 µM 2′7′-dichlorofluorescein diacetate (DCFH-DA) (Sigma) for 30 min at 37 °C under dark conditions. Then, the cells were rinsed two times with cooled 1X PBS and collected using 0.05% trypsin EDTA. Quantification of intracellular ROS was evaluated by a BD Accuri C6 flow cytometer system (BD Biosciences, Franklin Lakes, NJ, USA). The data were collected and analyzed using FCS 6 plus Research Edition software (De Novo Software, Pasadena, CA, USA).

### 2.11. Enzyme-Linked Immunosorbent Assay

HDF cells were seeded at a density of 1.5 × 10^5^ cells/mL in 35 mm cell culture plates to acquire treatment conditions after 24 h. After 72 h of UVB irradiation, cell supernatant was collected to measure the concentrations of MMP-1, MMP-3, and procollagen type 1 protein in media, which were estimated using commercially available ELISA kits according to the manufacturers’ instructions. Each sample was repeatedly analyzed twice.

### 2.12. Reverse Transcriptase (RT)-PCR

Cells were collected 24 h after sensitization with inducers. RNA was isolated by using TRIZOL reagent, according to the manufacture’s guidelines (Invitrogen Life Technologies, Carlsbad, CA, USA). An equal amount of RNA (3 µg) was reverse transcribed using a PCR premix (Bioneer Co., Daejoon, Korea), 0.5 µg/mL oligo-(dT)15 primer, and 0.5 µg/mL hexamer primer. The cDNA was resynthesized at 42 °C for 60 min and was incubated at 94 °C for 5 min to stop the reaction. PCR amplification was performed using a PCR premix (Bioneer) and designated primer pairs outlined in Supplementary Materials Table S2. Amplified products were observed by gel electrophoresis and detected by nucleic acid staining (NobleBio Inc., Gyeonggi, Korea) under UV illumination. GAPDH was used for normalization.

### 2.13. Western Blot

Cell lysates were merged in RIPA buffer (Sigma) for at least 1 h and centrifuged at 12,000 rpm in 15 min to obtain the total protein extract. To isolate the nucleoprotein, an NE-PER nuclear and cytoplasmic extraction reagents kit (Pierce) was used according to the manufacturer’s instructions. The protein concentration was calibrated using Bradford reagent (Bio-Rad). Homogenized proteins were separated by SDS-PAGE and transferred to a nitrocellulose membrane (Bio-rad). Transfer membranes were blocked in 5% skim milk or 5% BSA for 30 min. After several washing steps with 1X TBST, the primary membrane was added with primary antibodies overnight at 4 °C. After incubation with secondary antibody for 1 h, the protein bands were detected using chemiluminescence detection ECL reagents (Fujifilm, LAS-4000, Tokyo, Japan) and ImageMaster™ 17 2D Elite software, version 3.1 (Amersham Pharmacia Biotech, Piscataway, NJ, USA). β-actin and histone were used for the normalization of either the total protein extract or nuclear protein extract, respectively.

### 2.14. Statistical Analysis

The data were obtained by using the Statistical Analysis System GraphPad Prism 5 (GraphPad Software, San Diego, CA, USA). All experiments were conducted thrice, for three replications. Data are shown as mean ± standard deviation (SD). Significant differences between the different treatments were analyzed using a one-way analysis of variance followed by the Duncan’s test. The comparison between sample treatments and the control group were performed by using the Student’s t-tests. Statistical significance was set as follows: * *p* < 0.05, ** *p* < 0.01, and *** *p* < 0.001.

## 3. Results

### 3.1. Analysis of Chemical Contents of AC Extract

AC extract contained high concentrations of total phenols, flavonoids, and tannins, represented as 102.6 ± 0.61 mg gallic acid/g extract, 274.7 ± 1.16 mg quercetin/g extract, and 43.4 ± 1.33 mg catechin/g extract, respectively.

Tannic acid, chlorogenic acid, and apigenin were identified in AC leaf extract at the concentration of 18.83 ± 0.13, 0.94 ± 0.03, and 0.27 ± 0.01 mg/g, respectively (Supplementary Figure S1). The retention time of tannic acid, chlorogenic acid, and apigenin were 24.5, 14.3, and 33.4 min, respectively.

### 3.2. Antioxidative Activities of AC Extract

**Figure 1:**
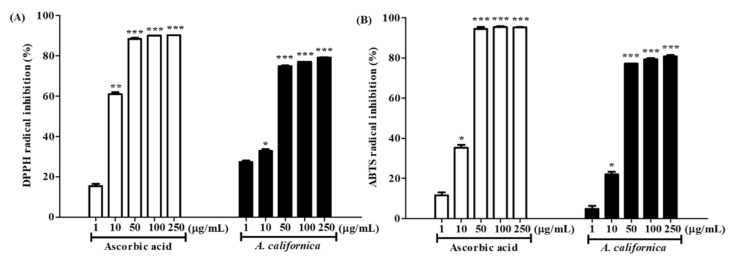
DPPH (**A**) and ABTS (**B**) inhibition of *A. californica* extract. The radical scavenging effect was presented as a percentage of that measured in the control group. Data are presented as the mean ± SD. *, **, and *** indicate the significant within-group differences (* *p* < 0.05 ** *p* < 0.01 and *** *p* < 0.001, respectively).

To evaluate the antioxidative activity of the AC extract, its radical scavenging effect was analyzed by DPPH and ABTS assays. The ascorbic acid and AC extract showed a dose-dependent inhibition of DPPH and ABTS radicals. As shown in Figure 1, the positive control, ascorbic acid, showed the scavenging effect on DPPH and ABTS radicals, with an IC_50_ value of 7.91 ± 0.33 μg/mL and 13.05 ± 0.28 μg/mL, respectively. AC extract also significantly suppressed DPPH radicals with an IC_50_ value of 20.72 ± 1.01 μg/mL, and ABTS radicals, with an IC_50_ value of 15.81 ± 0.78 μg/mL, suggesting that AC exhibited a potent antioxidative effect.

### 3.3. Cytotoxicity of AC Extract

**Figure 2:**
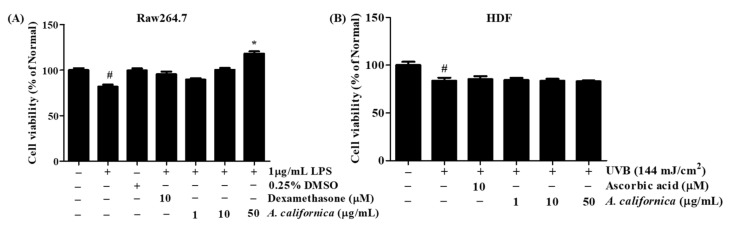
Effect *A. californica* extract on cell viability of Raw264.7 (**A**) and human dermal fibroblast (HDF) (**B**) cells. Data are presented as the mean ± SD. # and * indicate significant differences from the non-treated cells and induced groups, respectively. # *p* < 0.05 vs. the non-treated group. * *p* < 0.05 vs. the induced control.

The toxicity of AC on Raw264.7 and HDF cells was assessed by an MTT assay. As shown in Figure 2A, LPS treatment on Raw264.7 cells slightly decreased survival of cells by 28.2% when compared to the normal group; however, the supplement of 10 µM dexamethasone and AC recovered cell viability. When compared to LPS-treated control cells, at 50 µg/mL, the AC extract protected cells from apoptosis, enhancing living cells by 44.5%.

In Figure 2B, HDF cell survival of the irradiated control group was markedly reduced by 16.4% when compared to the untreated group. AC doses (1–50 µg/mL) did not show a significant effect on cell viability. Thus, AC at the concentrations of 1, 10 and 50 µg/mL were used for the cell treatment in further experiments.

### 3.4. AC Extract Regulates Inflammatory Response in Raw264.7 Cells

#### 3.4.1. Effect of AC Extract on NO Production in LPS-Induced Raw264.7 Cells

**Figure 3:**
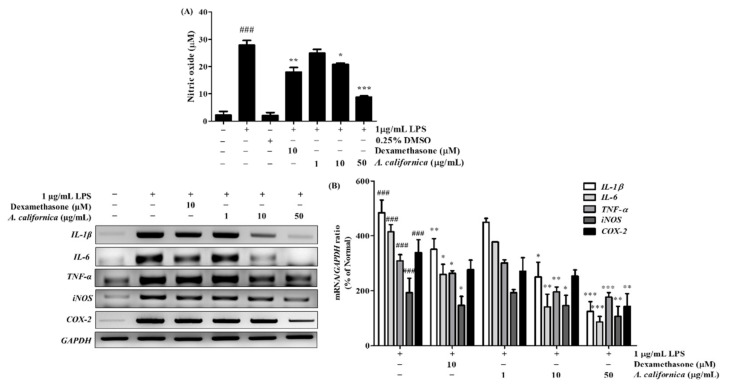
Effect of *A. californica* extract on NO production (**A**) and mRNA levels of IL-1β, IL-6, TNF- α, iNOS, and COX-2 (**B**) in LPS-induced Raw264.7 cells. Data are presented as the mean ± SD. # and * indicate significant differences from the non-treated cells and LPS-induced groups, respectively. ### *p* < 0.001 vs. the non-treated group. *, ** and *** *p* < 0.05, 0.01, and 0.001 vs. the LPS-induced control, respectively.

In photoaging, macrophages infiltrate to the site of skin damage to remove dead cells, releasing abundant types of inflammatory mediators which are important for wound healing after tissue injury. However, the uncontrollable release of these mediators may lead to inflammatory damage, with many undesirable immunological responses. NO overproduction is a hallmark of inflammatory responses. As shown in Figure 3A, in comparison to the untreated Raw264.7 cells, NO synthesis was increased by 1170.3% in the LPS-treated cells after 24 h. However, in AC-treated groups, NO secretion decreased by 25.1% and 68.3% at 10 and 50 µg/mL, respectively, when compared to LPS-treated controls. Dexamethasone also exhibited an inhibitory effect on NO production, which decreased by 35.4% when compared to the LPS-treated control.

#### 3.4.2. Effect of AC Extract on the mRNA Expression of IL-1β, IL-6, TNF-α, iNOS, and COX-2 in LPS-Induced Raw264.7 Cells

Inflammation triggers cellular signals that promote the production of inflammatory mediators, to recruit other immune cells to the site of infection. In response to LPS induction, the mRNA expression of cytokines such as IL-1β, IL-6, and TNF-α was accelerated by 384.2%, 314.7%, and 209.5%, respectively, when compared to untreated cells (Figure 3B). AC at 50 µg/mL reversed these changes, evidenced by the decline of IL-1β, IL-6, and TNF-α mRNA levels by 74.1%, 79.2%, and 42.7%, respectively, when compared to LPS-treated control cells. The AC treatment was more effective than the positive control, dexamethasone, which moderately inhibited IL-1β, IL-6, and TNF-α by 27.5%, 37.4%, and 14.7%, respectively.

Additionally, some inflammatory molecules, such as NO and prostaglandin E_2_, were synthesized by enzymes such as iNOS and COX-2, respectively. In Figure 3B, LPS sensitization promoted iNOS and COX-2 mRNA production by 93.6% and 239.1%, respectively, when compared to the normal group. However, the highest dose of AC effectively alleviated iNOS and COX-2 mRNA expression by 44.7% and 50.0%, respectively, when compared to the LPS-treated control.

### 3.5. AC Extract Protects HDF Cells from UVB Irradiation

#### 3.5.1. Effect of AC Extract on ROS Production in UVB-Irradiated HDF Cells

**Figure 4:**
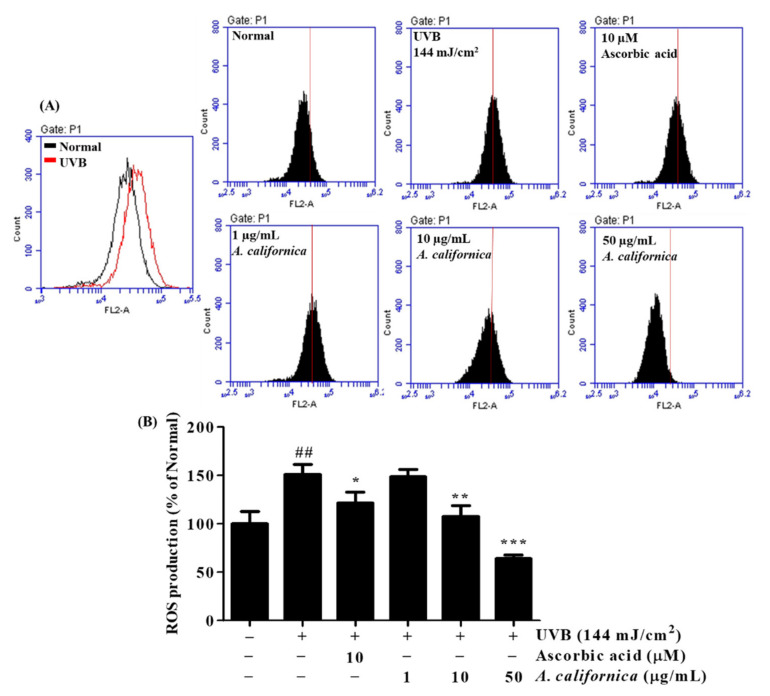
Effect of *A. californica* extract on levels of intracellular reactive oxygen species (ROS) in UVB-irradiated HDF cells. After 24 h of treatment, intracellular ROS level was measured. The number of cells is plotted versus the dichlorofluorescein fluorescence detected by the FL-2 channel (**A**). Results are presented as histograms (**B**). Data are presented as the mean ± SD. * indicate significant differences from the non-irradiated control and UVB-treated groups, respectively. ## *p* < 0.01 vs. the non-treated group. *, ** and *** *p* < 0.05, 0.01 and 0.001 vs. the UVB-treated control, respectively.

As shown in Figure 4, ROS formation sharply increased by 51.1% in the irradiated control group when compared with the non-treated group. However, the AC-treated group exhibited a significant decrease in ROS levels when compared with the irradiated control cells. In particularly, the treatment of 10 and 50 µg/mL AC lowered ROS formation by 28.9% and 57.7%, respectively. The positive control, ascorbic acid, also exhibited an inhibition of ROS production (by 19.6%).

#### 3.5.2. Effect of AC Extract on the Protein Secretion of MMP-1, MMP-3, and Procollagen Type I in UVB-Irradiated HDF Cells

**Figure 5:**
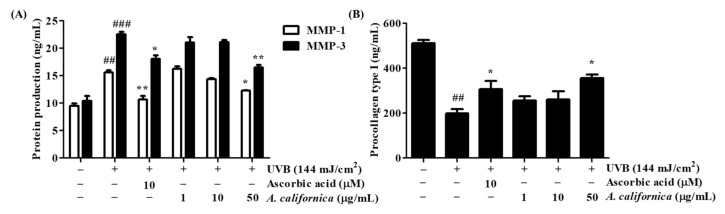
Effect of *A. californica* extract on the protein secretion of metalloproteinase 1 (MMP-1) and 3 (MMP-3) (**A**), and procollagen type I (**B**) in UVB-irradiated HDF cells. Data are presented as the mean ± SD. # and * indicate significant differences from the non-irradiated control and UVB-treated groups, respectively. ## and ### *p* < 0.01 and 0.001 vs. the non-treated group, respectively. * and ** *p* < 0.05 and 0.01 vs. the UVB-treated control, respectively.

To investigate the inhibitory effect of AC extract on collagen degradation, the study further quantified the secreted protein levels of MMP-1, MMP-3, and type I procollagen in irradiated cell culture supernatants by using ELISA kits. The exposure to UVB irradiation elevated MMP-1 and MMP-3 protein levels by 63.5% and 117.1%, respectively, meanwhile it also decreased procollagen type I production by 61.2% when compared to non-treated group (Figure 5). The treatment of AC resulted in a dose-dependent reversed effect, which inhibited the release of MMP-1 and MMP-3 by 21.2% and 26.8%, respectively, and promoted the synthesis of procollagen by 79.3% when compared to the irradiated control group.

#### 3.5.3. Effect of AC Extract on the mRNA and Protein Expression of MMP-1, TGF-β1, and Procollagen Type I in UVB-Irradiated HDF Cells

**Figure 6:**
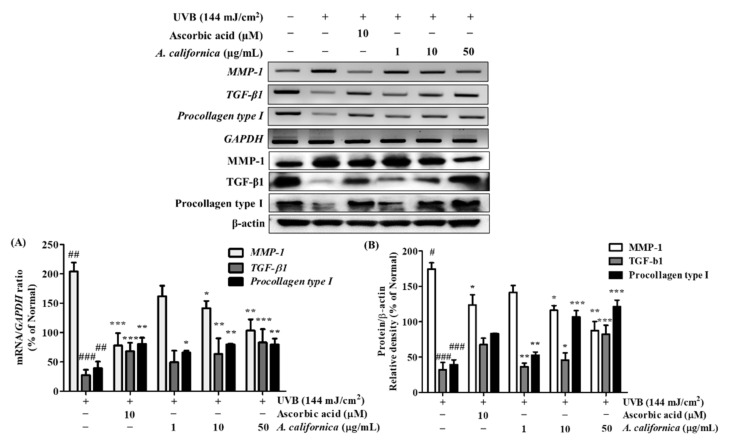
Effect of *A. californica* extract on mRNA (**A**) and protein (**B**) expression of MMP-1, TGF-β1, and procollagen type I in UVB-irradiated HDF cells. Data are presented as the mean ± SD. # and * indicate significant differences from the non-irradiated control and UVB-treated groups, respectively. ## and ### *p* < 0.01 and 0.001 vs. the non-treated group, respectively. *, ** and *** *p* < 0.05, 0.01 and 0.001 vs. the UVB-treated control, respectively.

As MMP-1 upregulation is a hallmark of photoaging, the mRNA expression studies indicated that irradiation raised the mRNA expression of MMP-1 by 104.1%; meanwhile, expression of the collagen synthesis activator, TGF-β1, and the collagen precursor procollagen type I were diminished by 72.4% and 60.8% when compared to the normal group, respectively (Figure 6A) AC extract reduced UVB-induced MMP-1 expression by 30.7% (at 10 μg/mL) and 49.4% (at 50 μg/mL). By contrast, AC (50 μg/mL) promoted TGF-β1 and procollagen type I expression by 200.0% and 102.5%, respectively, when compared with the irradiated control cells. This was comparable to the positive control, ascorbic acid (10 µM), which showed inhibition of the MMP-1 level by 62.2% and upregulation by 146.6% and 105.1%, for the TGF-β1 and procollagen type I levels, respectively.

Similarly, the protein expression study also indicated an upregulation of MMP-1 by 74.5%, and a consequently downregulated procollagen type I by 32.1% when compared to normal cells (Figure 6B) However, treatment with ascorbic acid and AC at 50 µg/mL reversed this trend, and diminished MMP-1 expression by 29.1% and 49.9% when compared to the irradiated control group, respectively. Furthermore, AC effectively promoted procollagen type I by 69.9% at 50 µg/mL. Under UVB irradiation, TGF-β1 was inhibited by 67.8% when compared to non-irradiated cells. However, AC recovered the expression level of TGF-β1 by 155.6% when compared to UVB-irradiated control group.

#### 3.5.4. Effect of AC Extract on MAPK/AP-1 Activation in UVB-Irradiated HDF Cells

**Figure 7:**
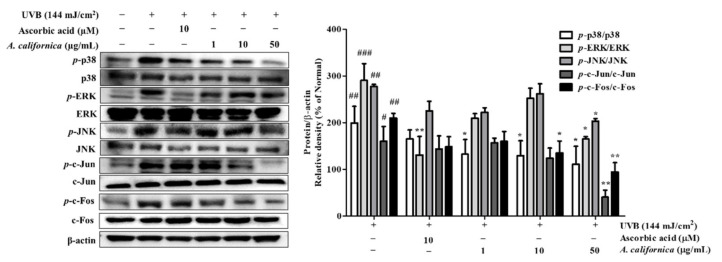
Effect of *A. californica* extract on the protein expression of phosphorylated MAPK/AP-1in UVB-irradiated HDF cells. Data are presented as the mean ± SD. # and * indicate significant differences from the non-irradiated control and UVB-treated groups, respectively. #, ##, and ### *p* < 0.05, 0.01 and 0.001 vs. the non-treated group, respectively. * and ** *p* < 0.05 and 0.01 vs. the UVB-treated control, respectively.

The downstream signaling that follows UVB includes the phosphorylation of the MAPK subunits: p38, ERK, and JNK. The effect of AC extract on MAPKs family members was studied in UVB-exposed dermal fibroblasts. As shown in Figure 7, UVB triggered an elevation of activated *p*-ERK, *p*-JNK, and *p*-p38. However, the treatment with AC extract dose-dependently reversed these changes. Supplement of 50 µg/mL AC extract suppressed the expression of *p*-p38, *p*-ERK, and *p*-JNK by 44.2%, 43.3%, and 26.7%, respectively.

Phosphorylation of MAPK subsequently activated the transcription factor c-Jun, which can reside in the nucleus to assemble with c-Fos to form the AP-1 transcription factor complex. AP-1 plays a pivotal role in MMP activation for collagen degradation. To further analyze the molecular mechanism of AC, protein levels of phosphorylated c-Fos and c-Jun were measured. As shown in Figure 7, UVB exposure upregulated the phosphorylation of c-Fos and c-Jun. However, treatment of cells with 50 µg/mL AC extract inhibited *p*-c-Fos and *p*-c-Jun levels by 55.0% and 74.8%, respectively.

#### 3.5.5. Effect of AC Extract on NRF2 Activation in UVB-Irradiated HDF Cells

**Figure 8:**
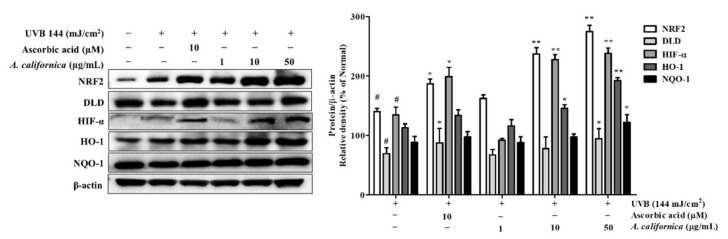
Effect of *A. californica* extract on the expression of NRF2, DLD, HIF-α, HO-1, and NQO-1 in UVB-irradiated HDF cells. Data are presented as the mean ± SD. # and * indicate significant differences from the non-irradiated control and UVB-treated groups, respectively. # *p* < 0.05 vs. the non-treated group. *and ** *p* < 0.05 and 0.01 vs. the UVB-treated control, respectively.

To assess the activation of the antioxidative system by AC treatment, the expression of the cytoprotective factor NRF2, as well as NRF2-regulated antioxidant proteins in UVB-exposed cells, were investigated. As shown in Figure 8, nuclear NRF2 protein expression was increased by UVB stimulation. Treatment of cells with 50 µg/mL AC extract increased NRF2 protein levels by 96.4% when compared with UVB control group. In addition, the reduction of DLD (by 30.3%) in UVB-irradiated cells, which is a flavoprotein enzyme of the TCA cycle-associated enzymes, was reversed by the AC treatment (increased by 36.0%). DLD not only associates with α-keto acid dehydrogenase, a regulator of ROS, but also metabolizes α-lipoic acid, which activates two cytoprotective proteins, NRF2 and HO-1. It was also found that AC treatment recovered the reduction in HIF1-α expression, which plays vital roles in the adaptive response to hypoxia, resulting in the increases of 68.7% (10 µg/mL) and 76.6% (50 µg/mL). This might result from NRF2 activation, which was reported to promote *HIF1A* gene expression and HIF1-α stability [30]. HIF1-α can translocate to the nucleus and transcribe hypoxic adaptation genes, such as HO-1 [13]. Moreover, HO-1 and NQO-1 protein expressions were significantly induced by the supplement of AC extract. As shown in Figure 8, the protein levels of HO-1 and NQO-1 were upregulated by 70.4% and 38.3% with 50 µg/mL AC treatment, respectively.

#### 3.5.6. Effect of AC Extract on NFATc1 Nuclear Translocation in UVB-Irradiated HDF Cells

**Figure 9:**
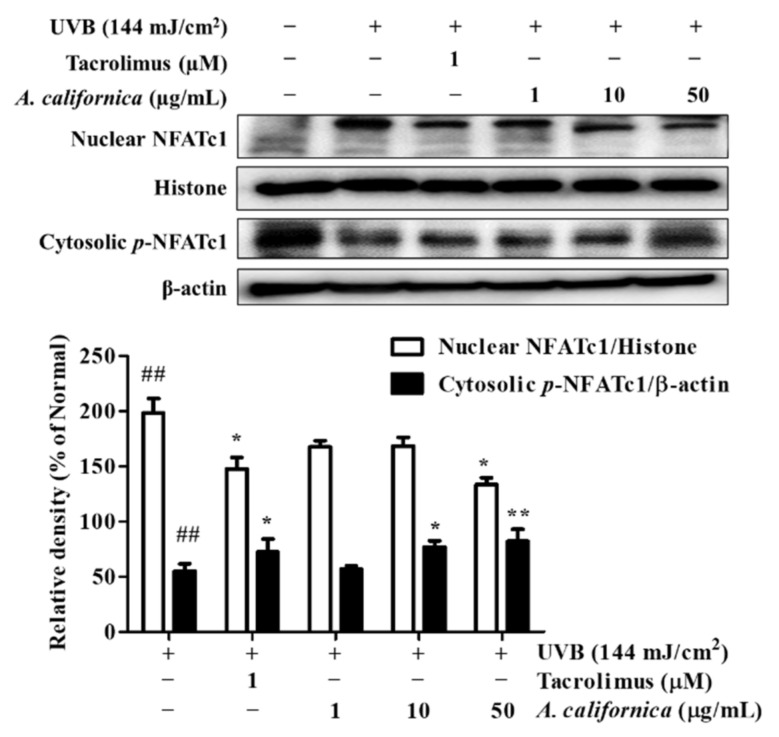
Effect of *A. californica* extract on the protein expression of nuclear NFATc1 and cytosolic phosphorylated NFATc1 in UVB-irradiated HDF cells. Data are presented as the mean ± SD. * indicate significant differences from the non-irradiated control and UVB-treated groups, respectively. ## *p* < 0.01 vs. the non-treated group. * and ** *p* < 0.05 and 0.01 vs. the UVB-treated control, respectively.

NFAT is strictly involved in the skin inflammatory response. Following dephosphorylation, activated NFAT translocates to the nucleus, initiating cytokine gene expression. Although NFAT has been well demonstrated for its role in immune systems, the merging effect of NFAT in photoaging has also been reported. Thus, NFATc1 protein expression was evaluated in UVB-irradiated HDF cells. As shown in Figure 9, UVB exposure induced NFATc1 expression by 98.2%, however, treatment with the calcineurin inhibitor tacrolimus and AC at 50 µg/mL inhibited NFATc1 by 25.6% and 32.7%, respectively, when compared to the normal group. Additionally, AC reversed the dephosphorylation of NFATc1 in the cytosol by 49.4%.

## 4. Discussion

AC, which is native to northwestern Mexico and southwestern USA, has been traditionally applied for treating bacterial infections and inflammation [23]. AC products, such as tea, tincture, and infusion, have also been utilized to alleviate the inflammatory injury of mucous membranes, swollen gums, and sore throats [22]. In addition, its dusting powder can be used externally for soaking inflamed or infected areas [29]. Furthermore, in vitro investigations have also demonstrated the biological effects of AC, such as its anti-bacterial [22,23], antioxidant [28], and anti-cancer activities [29]. However, despite the historical and modern use of this plant to treat acute inflammation of the skin, only a few studies have focused on the pharmacological effects and the mechanism underlying the effects of AC on chronic inflammation, such as in skin photoaging. Thus, in this study, we investigated the protective effects of AC treatment on LPS-stimulated murine macrophages and UVB-exposed human dermal fibroblasts.

UVB exposure leads to physical cutaneous tissue damage and chronic inflammation. The inflammatory response involves the infiltration of immune cells into the irradiated area, leading to the release of NO and pro-inflammatory mediators such as cytokines, chemokines, and prostaglandins. The control of inflammatory signaling molecules is essential for avoiding inflammatory injuries. The current study found that the use of AC extract effectively downregulated the secretion of NO by approximately two-thirds, and the gene expression of inflammatory mediators, including IL-1β, IL-6, TNF-α, COX-2, and iNOS, by about half when compared with the levels of the LPS control. Noticeably, treatment with a high concentration of AC resulted in a reduction in the mRNA expression of inflammatory molecules, showing superior effects when compared with the anti-inflammatory drug dexamethasone, a corticosteroid with severe side effects such as rash, acne, allergic dermatitis, and impaired wound healing [31].

To combat skin aging, the key strategy is to sustain the synthesis of collagen, the main constituent of the ECM, contributing to 75% of the dry weight of the skin and providing integrity and elasticity [32]. In this study, increases in collagenases such as MMP-1 and -3 under UVB irradiation were identified, further degrading type I collagen, which constitutes 80% to 90% of the total collagen of human skin [33]. However, AC treatment significantly reversed this change by inhibiting the expression of MMP-1 by about 50% at both mRNA and protein levels in UVB-irradiated dermal fibroblasts. In addition to the downregulation of collagen degradation, AC also led to the upregulation of a procollagen inducer, TGF-β1. A high dose of AC increased the mRNA and protein expression of TGF-β1 by over 100%, which was comparable to the effect of the positive control, ascorbic acid (10 µM), which is a cofactor in the biosynthesis of procollagen and elastin. Furthermore, collagen homeostasis was significantly influenced by the activation of the transcription factor AP-1, which not only initiates the mRNA expression of several collagenases, including MMP-1, -3, and -9, but also depresses the production of procollagens (precursors of collagens) by downregulating the gene expression of procollagen type I. Under UVB irradiation, the activation of AP-1 is mediated via MAPK phosphorylation, which is induced by high intracellular ROS levels. The results from this study indicated a three-fold reduction in ROS formation and the protein expression of phosphorylated MAPK subunits in AC-treated cells, consequently suppressing MMP expression under UVB induction.

A study by Carmen et al. also reported high antioxidant levels in the stems and leaves of AC upon measuring lipid peroxidation and scavenged free radicals after arsenic exposure [34]. AC exhibited a low level of thiobarbituric acid reactive substances (TBARS), typical oxidative stress markers, and a higher level of scavenged DPPH radicals under arsenic-exposed conditions. This is consistent with the results from the current study, in which AC showed inhibitory effects on DPPH and ABTS radicals at IC_50_ values of 20.72 ± 1.01 μg/mL and 15.81 ± 0.78 μg/mL, respectively. Additionally, one study revealed activation of the antioxidant defense system NRF2 under UVB irradiation [35]. The transcription factor NRF2 might be switched on to detoxify excessively produced ROS in irradiated cells [35]. As NRF2 is required for the transcription of ROS quenching proteins, such as NQO1 and HO-1, it was found that there was an increase in nuclear-resident NRF2 in UVB-exposed cells [36]. Interestingly, AC treatment induced a sharp increase in the nuclear translocation of NRF2, which was even higher than that for the well-known antioxidant ascorbic acid, a common constituent of skincare products. This resulted in the mass production of detoxifying proteins such as NQO1 and HO-1 in the AC-treated cells. In addition, it was found that a high dose of AC restored the expression of DLD protein, which is not only associated with α-keto acid dehydrogenase, a regulator of ROS, but also metabolizes α-lipoic acid, which activates two cytoprotective proteins, NRF2 and HO-1. NRF2 is also involved in the upregulation of the transcription factor HIF1-α, which is required for the transcription of HO-1 in the hypoxic response [37]. The removal of excess ROS in irradiated cells is has been indicated to prevent further DNA damage and the activation of AP-1, which upregulates collagenase MMPs. Moreover, genetic or pharmacological NRF2 activation downregulated the mRNA expression of interleukin (IL)-6 and IL-1β, and cyclooxygenase (COX)-2 in mice after UV exposure [38]. Additionally, a human study revealed that topical applications of the Nrf2 activator sulforaphane alleviated the level of solar-induced skin erythema, which indicates a high risk of skin cancer [38]. Thus, the antioxidant AC was suggested to be an effective contributor to preventing the photodamage of skin.

In this study, the high concentrations of chemical components such as phenols, flavonoids, and tannins found in AC extract were suggestive of its biological activities. In particular, the HPLC results from the current study identified three phytochemicals in AC extract: tannic acid, chlorogenic acid, and apigenin. Tannic acid decreased the levels of ROS and pro-inflammatory cytokines in phorbol 12-myristate 13-acetate (PMA)- or H_2_O_2_-induced Raw264.7 cells; meanwhile, in an in vivo zymosan-induced peritonitis mouse model, tannic acid alleviated neutrophil recruitment and pro-inflammatory cytokines [39]. Moreover, as a strong antioxidative agent that attenuates ROS formation and NADPH oxidase activation, and reduces the activity of the endogenous antioxidant defense system, tannic acid prevented photodamage by reducing MMP-1 production in UVB-irradiated L929 fibroblasts [40]. Other active components found in AC, such as chlorogenic acid and apigenin, also exhibited anti-inflammatory effects, which reduced inflammatory mediators (IL-1β, TNF-α, iNOS, NO, and COX-2) in LPS-induced Raw264.7 macrophages [41,42]. Moreover, apigenin alleviated apoptosis in UVB-irradiated keratinocytes by activating antiapoptotic protein B-cell lymphoma 2 (Bcl-2) [43] and inhibiting COX-2 [44]. Chlorogenic acid was also reported to prevent DNA damage in UVB-exposed human keratinocytes, resulting from ROS reduction and downregulation of the pro-apoptotic marker cleaved caspase-3 [45]. Thus, the synergistic effects of these identified active components might contribute to the anti-inflammatory and photoprotective effects of AC extract.

Moreover, the key immune regulator NFATc1 was also found to be expressed in non-immune cells, namely, dermal fibroblasts, under UVB irradiation. This can be explained by the reciprocal effect between the overproduction of ROS and intracellular calcium influx [46]. As intracellular calcium concentration is elevated, calcineurin dephosphorylates NFAT to promote the nuclear localization of NFAT [15]. NFAT was reported to coordinate with c-Jun to initiate the transcription of collagenase MMPs [47,48]. Furthermore, targeting NFAT resulted in the downregulation of COX-2, which is involved in carcinogenesis [19]. In the current study, AC treatment prevented the translocation of NFATc1 in UVB-irradiated dermal fibroblasts by upregulating phosphorylated NFATc1, which might subsequently reduce the activation of the AP-1 complex in the nucleus. This results in lower MMP-1 mRNA expression in AC-treated cells, further supporting the restoration of collagen.

## 5. Conclusions

This study was conducted to assess the protective effect of AC against photoaging in an in vitro model. The results indicated that AC treatment might regulate the activation of macrophages by reducing the secretion of NO and pro-inflammatory cytokines, consequently alleviating the inflammatory response. In addition, AC treatment restored the synthesis of procollagen type I in UVB-irradiated dermal fibroblasts by inhibiting MMP expression and activating the collagen inducer TGF-β1. This further prevented the degradation of ECM components during UVB exposure, thus reducing the formation of aging phenotypes. Besides, AC acted as a strong source of antioxidants, which prevented oxidative imbalance by inhibiting ROS formation and activating the cytoprotective NRF2. The relief of cellular oxidative stress can alleviate macrophage infiltration and MAPK activation, thus minimizing skin damage caused by the overexpression of MMP. In particular, the study indicated that NFATc1, a central regulator of the immune system, might be a promising target for efforts to counter photoaging. The inhibition of the nuclear localization of NFATc1 by AC might decrease the expression of MMP genes, as well as genes encoding pro-inflammatory cytokines such as IL-6 and TNF-α.

In summary, the multiple functions of AC suggested that it is an effective alternative therapeutic candidate for skin diseases, especially in the photodamage of skin. Nevertheless, there is a need for the further evaluation of the biological effects of AC and its active compounds on in vivo models and in clinical trials.

## Data Availability

The data presented in this study are available in this paper.

## Supplementary Materials

The following are available online at https://www.mdpi.com/article/10.3390/antiox10121882/s1, Table S1: HPLC method for the identification of AC extract, Table S2: Oligonucleotide primers used for RT-PCR, Figure S1: HPLC analysis of standard tannic acid (A) and *A. californica* extract (B) at 272 nm. HPLC analysis of standard chlorogenic acid and apigenin (C) and *A. californica* extract (D) at 360 nm.
