# Supplementary material for: Anemopsis californica Attenuates Photoaging by Regulating MAPK, NRF2, and NFATc1 Signaling Pathways
Source: Antioxidants (Basel). 2021 Nov 25;10(12):1882. doi: 10.3390/antiox10121882 (PMC8698643; doi:10.3390/antiox10121882)
Supplement: Supplementary file 1 [file antioxidants-10-01882-s001.zip › antioxidants-1469110-supplementary.pdf]

Table S1. HPLC method for the identification of AC extract

Elution condition	Tannic acid	Chlorogenic acid and apigenin
**Mobile phase A**	0.1% formic acid in DW	0.1% formic acid in DW
**Mobile phase B**	acetonitrile	0.1% formic acid in acetonitrile
**Gradient linear**	8% B to 95% B	5% B to 40% B
**Retention time**	40 mins	50 mins
**UV detection**	272 nm	360 nm

Table S2. Oligonucleotide primers used for RT-PCR

NCBI accession code	Primer [a]		Sequences (5′-3′)
XM_011526432.2	Human GAPDH	Sense	ACC ACA GTC CAT GCC ATC AC
		Antisene	CCA CCA CCC TGT TGC TGT AG
NM_001145938.2	Human MMP-1	Sense	ATT CTA CTG ATA TCG GGG CTT TGA
		Antisene	ATG TCC TTG GGG TAT CCG TGT AG
NM_000088.4	Human procollagen type I	Sense	CTC GAG GTG GAC ACC ACC CT
		Antisene	CAG CTG GAT GGC CAC ATC GG
NM_000660.7	Human TGFβ-1	Sense	GCC CTG GAC ACC AAC TAT TGC
		Antisene	GCT GCA CTT GCA GGA GCG CAC
XM_036165840.1	Rat GAPDH	Sense	TGA TGA CAT CAA GAA GGT GGT GAA G
		Antisene	TCC TTG GAG GCC ATG TAG GCC AT
NM_001313922.1	Rat iNOS	Sense	CCT CCT CCA CCC TAC CAA GT
		Antisene	CAC CCA AAG TGC TTC AGT CA
NM_011198.4	Rat COX-2	Sense	ACT CAC TCA GTT TGT TGA GTC ATT C
		Antisene	TTT GAT TAG TAC TGT AGG GTT AAT G
NM_008361.4	Rat IL-1β	Sense	TGC AGA GTT CCC CAA CTG GTA CAT C
		Antisene	GTG CTG CCT AAT GTC CCC TTG AAT C
NM_001314054.1	Rat IL-6	Sense	CTG CAA GAG ACT TCC ATC CAG
		Antisene	AGT GGT ATA GAC AGG TCT GTT GG
NM_001278601.1	Rat TNF-α	Sense	TCT CAT CAG TTC TAT GGC CC
		Antisene	GGG AGT AGA CAA GGT ACA AC

[a]: Primer design based on the NCBI/Primer-BLAST tool with standard parameters

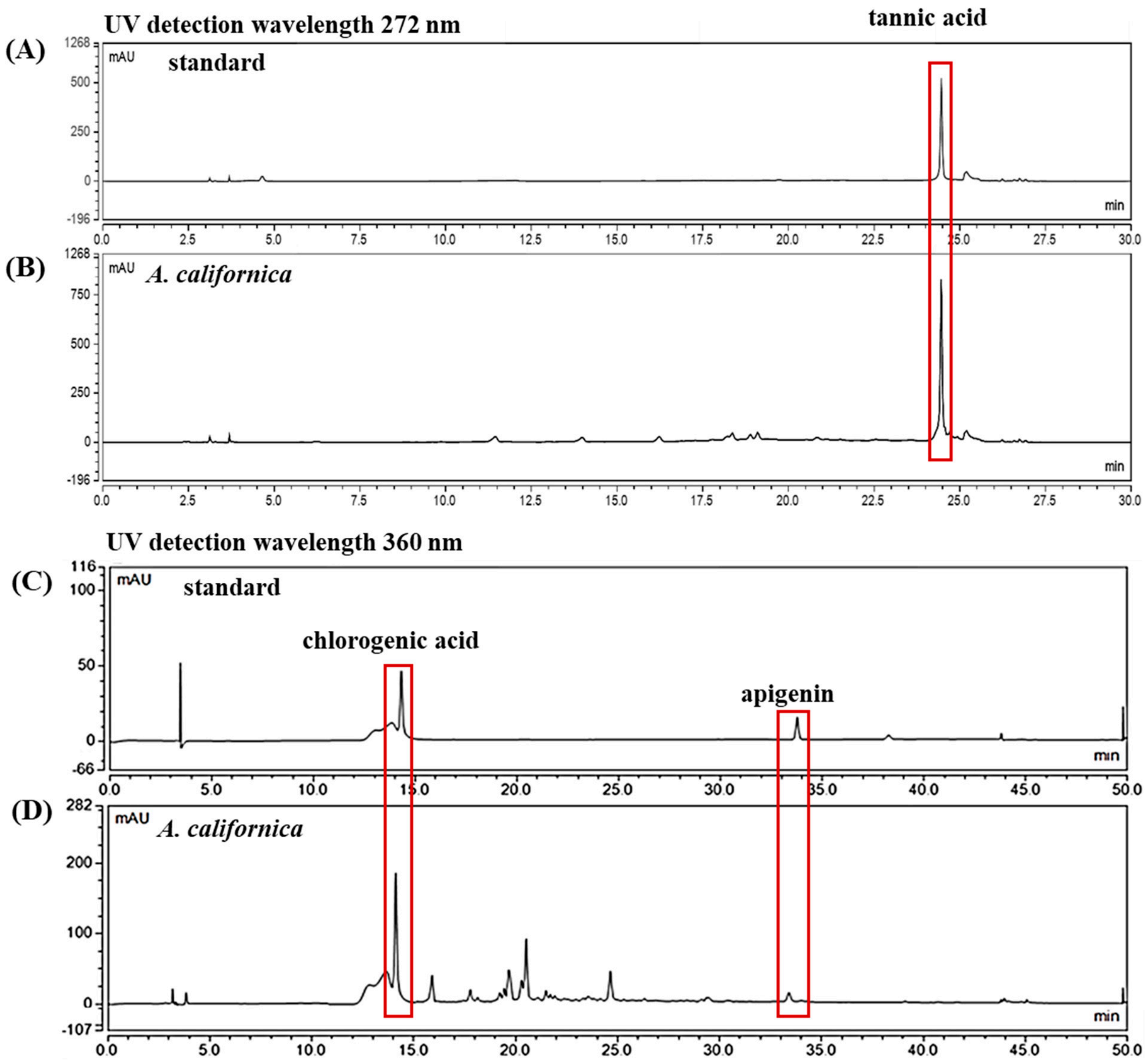

Figure S1. HPLC analysis of standard tannic acid (A) and *A. californica* extract (B) at 272 nm. HPLC analysis of standard chlorogenic acid and apigenin (C) and *A. californica* extract (D) at 360 nm.
